# Between the algorithm and clinical reasoning

**DOI:** 10.1590/2175-8239-JBN-2025-E019en

**Published:** 2025-11-28

**Authors:** Caio Oliveira Bastos, Kleyton de Andrade Bastos

**Affiliations:** 1 Fundação Oswaldo Ramos, Hospital do Rim e Hipertensão, São Paulo, SP, Brazil.; 2 Universidade de São Paulo, Faculdade de Medicina, Centro de Desenvolvimento em Educação Médica, São Paulo, SP, Brazil.; 3 Universidade Federal de Sergipe, Departamento de Medicina, Aracaju, SE, Brazil.

Generative artificial intelligence (AI), powered by large language models (LLMs), has emerged as one of the most transformative innovations of our era. These systems, trained on vast volumes of text and data, are capable of processing and generating human language with unprecedented fluency. Although powerful models such as GPT-3 had existed since 2020, it was the release of the public ChatGPT interface (OpenAI) in November 2022 that became the catalyst of a revolution^1^. By broadening access to AI, ChatGPT accelerated its adoption across different sectors at an unprecedented pace—culminating in the launch of GPT-5 in August 2025. In this context of rapid transformation, medicine, and specifically nephrology, has begun to explore the real impact of this tool. Feitosa et al.^2^ recently published in this journal the data of a pioneering national study that evaluated the performance of GPT-3.5 and GPT-4 in solving 411 nephrology residency examination questions administered between 2010 and 2024.

The design of this pilot study involved a direct comparison between the two versions of the model within four key domains: hydroelectrolytic and acid-base disorders, tubulointerstitial diseases, chronic kidney disease (CKD), and glomerular diseases. The results showed that GPT-4 achieved an overall accuracy of 79.8%, significantly higher than GPT-3.5’s 56.3% (p < 0.001), with consistent superiority across all subtopics. Furthermore, GPT-4 performed better on text-based questions (81.5%), but showed limitations on image-based questions (54.5%). The findings reported by the authors suggest that GPT-4 may be a promising tool for medical education, simulating clinical scenarios and assisting in the development of diagnostic reasoning^2^.

These results echo what international literature has demonstrated. Studies comparing the performance of LLMs with that of physicians on certification examinations and multiple international licensing exams have shown that GPT-4 reached accuracy levels above 90%^3,4,5^. Interestingly, its performance varied between exams, which may reflect not only differences in test design but also cultural and curricular particularities that shape how medical knowledge is assessed in different countries^5^. This underscores the importance of local validation studies, such as the study by Feitosa et al., which ensure the contextual relevance of technology assessment.

The educational implications are vast. The true value of LLMs lies not in their role as repositories of correct answers, but in their potential as interactive learning partners. More broadly, AI can support educational efforts by enhancing preclinical and clinical learning, assisting diagnostic processes, making assessments more efficient, reducing faculty workload, providing feedback, and generating insights to guide curricula and evidence-based policies^6^. In specialties with a high cognitive load, such as nephrology, AI can act as a catalyst of knowledge, structuring and personalizing the educational experience. Instead of focusing on memorization, learners can use AI platforms to mitigate this load through simulations and adaptive scenarios^7^. This type of interaction, simultaneously scalable and personal, allows them to test hypotheses and refine clinical reasoning in a safe, controlled environment, and has been associated among medical students with improvements in diagnostic accuracy and problem-solving skills^8^. In this sense, LLMs can serve as a supportive layer in the educational infrastructure of the specialty, assisting both learners and teachers. AI, therefore, if carefully used, does not replace the educator but complements them, allowing medical education to shift from “what” to “how”.

However, human–AI collaboration is not without paradoxes. While AI can increase the rate of solving complex cases, it can also induce error. A recent study revealed that although AI improved overall performance, some specialists missed questions they had previously answered correctly, precisely after being influenced by the algorithm’s incorrect suggestion^9^. Furthermore, excessive technological dependence can lead to “deskilling” (loss of acquired skills) and “never-skilling” (failure to develop essential competencies)^10^. These aspects reinforce the central thesis: AI is a powerful tool, but one that requires constant critical oversight, acting as a copilot rather than as an autopilot.

The reflection goes beyond education. In clinical practice, AI systems are already capable of identifying patients with CKD and stratifying its progression, predicting the risk of acute kidney injury, and optimizing compatibility in kidney transplantation^11,12^. The increasing accuracy of these models in factual knowledge tasks, especially those based on clinical guidelines, suggests that they are approaching expert-level performance. With the reduction of intrinsic errors, failures in responses will likely reflect more the ambiguities in question formulation than the system’s limitations. In this scenario, a fundamental question arises: what will be the physician’s role when algorithms assume responsibility for memory and knowledge retrieval? The future challenges us to cultivate essentially human competencies such as critical thinking, decision-making in uncertain contexts, empathy, and leadership.

The integration of LLMs into health professions education and medical practice is no longer a speculative possibility, but an inevitable trajectory. The question is not “if”, but “how” we will implement this integration in an ethical, safe, regulated, and ultimately patient-centered manner. The challenge ahead is to ensure that AI incorporation occurs critically and strategically, maximizing benefits for education and clinical care while minimizing risks. Only then will we be able to transform the potential of the algorithm into concrete benefits for the community.

## Data Availability

No new data were generated or analyzed in this study.
