# Supplementary material for: Between the algorithm and clinical reasoning
Source: J Bras Nefrol. 2025 Nov 28;47(4):e2025E019. doi: 10.1590/2175-8239-JBN-2025-E019en (PMC12704871; doi:10.1590/2175-8239-JBN-2025-E019en)
Supplement: Translation

# Translation: Entre o algoritmo e o raciocínio clínico

**DOI:** 10.1590/2175-8239-JBN-2025-E019pt

**Authors:** Caio Oliveira Bastos, Kleyton de Andrade Bastos

**Affiliations:** 1 Fundação Oswaldo Ramos, Hospital do Rim e Hipertensão, São Paulo, SP, Brasil.; 2 Universidade de São Paulo, Faculdade de Medicina, Centro de Desenvolvimento em Educação Médica, São Paulo, SP, Brasil.; 3 Universidade Federal de Sergipe, Departamento de Medicina, Aracaju, SE, Brasil.

A inteligência artificial (IA) generativa, impulsionada por modelos de linguagem de grande porte (LLMs), desponta como uma das inovações mais transformadoras da nossa era. Esses sistemas, treinados em vastos volumes de texto e dados, são capazes de processar e gerar linguagem humana com uma fluidez sem precedentes. Embora modelos poderosos, como o GPT-3, já existissem desde 2020, foi o lançamento da interface pública do ChatGPT (OpenAI), em novembro de 2022, que se tornou o catalisador de uma revolução^1^. Ao democratizar o acesso à IA, o ChatGPT acelerou, de forma inédita, sua adoção em diferentes setores, evoluindo em ritmo vertiginoso - culminando no lançamento do GPT-5 em agosto de 2025. É nesse cenário de rápida transformação que a medicina, e especificamente a nefrologia, começa a explorar o impacto real dessa ferramenta. Feitosa et al.^2^ publicaram recentemente, neste periódico, os dados de um estudo pioneiro no país, no qual avaliaram o desempenho das versões GPT-3.5 e GPT-4 na resolução de 411 questões de exames de residência em nefrologia, aplicados entre 2010 e 2024.

O desenho deste estudo piloto envolveu a comparação direta entre as duas versões do modelo em quatro domínios principais: distúrbios hidroeletrolíticos e acidobásicos, doenças tubulointersticiais, doença renal crônica (DRC) e doenças glomerulares. Os resultados mostraram que o GPT-4 obteve uma acurácia geral de 79,8%, significativamente superior aos 56,3% do GPT-3.5 (p < 0,001), com vantagem consistente em todos os subtemas. Além disso, o GPT-4 apresentou desempenho superior em questões textuais (81,5%), mas mostrou limitações em questões com imagens (54,5%). Os achados descritos pelos autores sugerem que o GPT-4 pode representar uma ferramenta promissora para o ensino médico, simulando cenários clínicos e auxiliando no desenvolvimento do raciocínio diagnóstico^2^.

Os resultados ecoam o que a literatura internacional vem demonstrando. Estudos comparando o desempenho de LLMs com o de médicos em exames de certificação e em múltiplos exames de licenciamento internacionais demostraram que o GPT-4 atingiu índices de acerto superiores a 90%^3–5^. Curiosamente, seu desempenho variou entre os exames, o que pode refletir não apenas diferenças no formato das provas, mas também particularidades culturais e curriculares que moldam a forma como o conhecimento médico é avaliado em diferentes países^5^. Isso ressalta a importância de estudos de validação locais, como o brasileiro, que garantem a relevância contextual da avaliação da tecnologia.

As implicações educacionais são vastas. O verdadeiro valor dos LLMs reside não em sua capacidade de atuar como um gabarito de respostas, mas em seu potencial como parceiros interativos de aprendizagem. De maneira mais ampla, a IA pode apoiar as ações educacionais ao potencializar a aprendizagem pré-clínica e clínica, auxiliar processos diagnósticos, tornar as avaliações mais eficientes, reduzir a carga docente, oferecer feedback e gerar *insights* para direcionar currículos e políticas baseadas em evidências^6^. Em especialidades com alta carga cognitiva, como a nefrologia, a IA pode atuar como um catalisador do conhecimento, estruturando e personalizando a experiência educacional. Em vez de focar na memorização, os aprendizes podem utilizar plataformas de IA para mitigar essa carga, por meio de simulações e cenários adaptativos^7^. Esse tipo de interação, ao mesmo tempo escalável e personalizada, permite que eles testem hipóteses e aprimorem o raciocínio clínico em um ambiente seguro e controlado, e tem sido associada, em estudantes de medicina, a melhorias na precisão diagnóstica e nas habilidades de resolução de problemas^8^. Nesse sentido, os LLMs podem funcionar como uma camada auxiliar da infraestrutura educacional na especialidade, apoiando tanto aprendizes quanto professores. Dessa forma, se utilizada com cautela, a IA não substitui o educador, mas o complementa, permitindo que a educação médica se desloque do “o quê” para o “como”.

No entanto, a colaboração humano-IA não é isenta de paradoxos. Se, por um lado, a IA pode aumentar a taxa de resolução de casos complexos, por outro, também pode induzir ao erro. Um estudo recente revelou que, embora a IA tenha melhorado o desempenho geral, alguns especialistas erraram questões que haviam acertado anteriormente, justamente após serem influenciados por uma sugestão incorreta do algoritmo^9^. Além disso, a dependência tecnológica excessiva pode levar ao *deskilling* (perda de habilidades adquiridas) e ao *never-skilling* (falha em desenvolver competências essenciais)^10^. Estes aspectos reforçam a tese central: a IA é uma ferramenta poderosa, mas exige supervisão crítica constante, devendo ocupar o papel de copiloto, e não de piloto automático.

A reflexão vai além do ensino. Na prática clínica, sistemas de IA já demonstram capacidade de identificar pacientes com DRC e estratificar a progressão da doença, prever o risco de injúria renal aguda e otimizar a compatibilidade em transplantes renais^11,12^.

A crescente precisão desses modelos em tarefas de conhecimento factual, especialmente aquelas baseadas em diretrizes clínicas, sugere que eles estão se aproximando de um desempenho em nível de especialista. Com a redução dos erros intrínsecos, as falhas nas respostas provavelmente refletirão mais as ambiguidades na formulação das perguntas do que as limitações do próprio sistema. Diante desse cenário, surge uma questão fundamental: qual será o papel do médico quando os algoritmos assumirem a responsabilidade pela memória e pela recuperação de conhecimento? O futuro nos desafia a cultivar competências essencialmente humanas, como pensamento crítico, tomada de decisão em contextos incertos, empatia e liderança.

A integração dos LLMs na educação das profissões da saúde e na prática médica deixou de ser uma possibilidade especulativa para se tornar uma trajetória inevitável. A questão que se impõe não é “se”, mas “como” faremos essa integração de maneira ética, segura, regulamentada e, em última análise, centrada no paciente. O desafio à nossa frente é garantir que a incorporação da IA ocorra de maneira crítica e estratégica, maximizando os benefícios para a educação e o cuidado clínico e minimizando os riscos. Somente assim poderemos transformar o potencial do algoritmo em benefício concreto para a comunidade.

## Data Availability

Nenhum dado novo foi gerado ou analisado neste estudo.
